# Reduced field-of-view DWI based on deep learning reconstruction improving diagnostic accuracy of VI-RADS for evaluating muscle invasion

**DOI:** 10.1186/s13244-024-01686-9

**Published:** 2024-06-09

**Authors:** Xinxin Zhang, Xiaojuan Xu, Yichen Wang, Jie Zhang, Mancang Hu, Jin Zhang, Lianyu Zhang, Sicong Wang, Yi Li, Xinming Zhao, Yan Chen

**Affiliations:** 1 Department of Diagnostic Radiology, National Cancer Center/National Clinical Research Center for Cancer/Cancer Hospital, Chinese Academy of Medical Sciences and Peking Union Medical College, Beijing, 100021 China; 2 GE Healthcare, MR Research China, Daxing district Tongji south road No1, Beijing, 100176 China; 3 School of Statistics and Mathematics, Nanjing Audit University, Nanjing, 211815 China

**Keywords:** Urinary bladder neoplasms, MRI, Deep learning reconstruction, Reduced field-of-view DWI, VI-RADS

## Abstract

**Objectives:**

To investigate whether reduced field-of-view (rFOV) diffusion-weighted imaging (DWI) with deep learning reconstruction (DLR) can improve the accuracy of evaluating muscle invasion using VI-RADS.

**Methods:**

Eighty-six bladder cancer participants who were evaluated by conventional full field-of-view (fFOV) DWI, standard rFOV (rFOV_STA_) DWI, and fast rFOV with DLR (rFOV_DLR_) DWI were included in this prospective study. Tumors were categorized according to the vesical imaging reporting and data system (VI-RADS). Qualitative image quality scoring, signal-to-noise ratio (SNR), contrast-to-noise ratio (CNR), and ADC value were evaluated. Friedman test with post hoc test revealed the difference across the three DWIs. Receiver operating characteristic analysis was performed to calculate the areas under the curve (AUCs).

**Results:**

The AUC of the rFOV_STA_ DWI and rFOV_DLR_ DWI were higher than that of fFOV DWI. rFOV_DLR_ DWI reduced the acquisition time from 5:02 min to 3:25 min, and showed higher scores in overall image quality with higher CNR and SNR, compared to rFOV_STA_ DWI (*p* < 0.05). The mean ADC of all cases of rFOV_STA_ DWI and rFOV_DLR_ DWI was significantly lower than that of fFOV DWI (all *p* < 0.05). There was no difference in mean ADC value and the AUC for evaluating muscle invasion between rFOV_STA_ DWI and rFOV_DLR_ DWI (*p* > 0.05).

**Conclusions:**

rFOV DWI with DLR can improve the diagnostic accuracy of fFOV DWI for evaluating muscle invasion. Applying DLR to rFOV DWI reduced the acquisition time and improved overall image quality while maintaining ADC value and diagnostic accuracy.

**Critical relevance statement:**

The diagnostic performance and image quality of full field-of-view DWI, reduced field-of-view (rFOV) DWI with and without DLR were compared. DLR would benefit the wide clinical application of rFOV DWI by reducing the acquisition time and improving the image quality.

**Key Points:**

- Deep learning reconstruction (DLR) can reduce scan time and improve image quality.
- Reduced field-of-view (rFOV) diffusion-weighted imaging (DWI) with DLR showed better diagnostic performances than full field-of-view DWI.
- There was no difference of diagnostic accuracy between rFOV DWI with DLR and standard rFOV DWI.

**Graphical Abstract:**

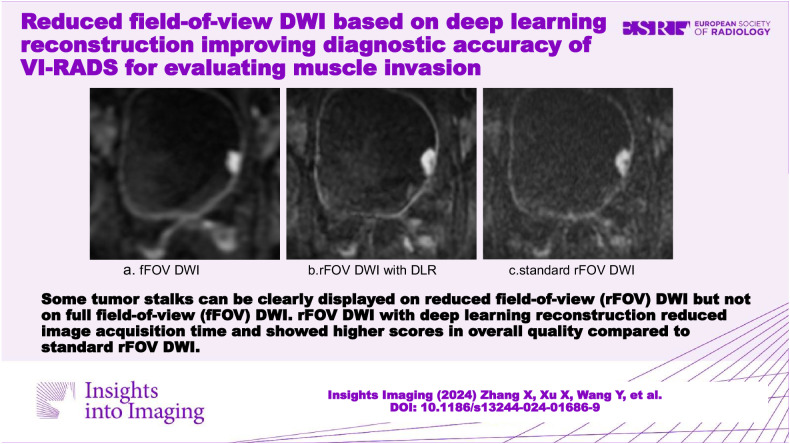

## Introduction

Bladder cancer is the 10th most commonly diagnosed cancer worldwide, with approximately 573,000 new cases and 213,000 deaths [1]. Distinguishing between muscle-invasive bladder cancer (MIBC) and non-muscle-invasive bladder cancer (NMIBC) is crucial for selecting the appropriate therapeutic approach. In this regard, multiparametric MRI has demonstrated exceptional capabilities in effectively determining the presence of muscle invasion in bladder cancer [2]. Based on bladder MRI, the vesical imaging reporting and data system (VI-RADS) scoring system was introduced to standardize the scanning protocol and reporting criteria to evaluate muscle invasion in 2018 [3]. Numerous investigations have demonstrated the promising performance of VI-RADS in evaluating muscle invasion in bladder cancer.

Diffusion-weighted imaging (DWI) is a routine sequence in bladder magnetic resonance imaging (MRI) and plays a critical role in determining the VI-RADS score [3, 4]. Previous studies demonstrated that DWI could clearly detect stalks of papillary bladder tumors and improved accuracy in distinguishing between MIBC and NMIBC [5, 6]. Currently, the widely used DWI is full field-of-view (FOV) single-shot echo-planar imaging, which is susceptible to susceptibility artifacts and image distortion. Moreover, the spatial resolution of this technique is restricted. The reduced FOV (rFOV) DWI (FOV optimized and constrained undistorted single-shot [FOCUS] DWI in GE, ZOOMit in Siemens, and zonal oblique multislice [ZOOM] in Philips) is a specific imaging technique that addresses these common problems in single-shot (SS) DWI. rFOV DWI achieves focused excitation of a reduced FOV in phase-encoding direction by using a 2D spatially selective echo-planar radiofrequency pulse and a 180 refocus pulse [7, 8]. This results in higher spatial resolution, reduced artifacts, and blurring while being less sensitive to field susceptibility and long-term eddy currents. rFOV DWI has been successfully applied in various anatomical regions such as the rectum, prostate, pancreas, and breast [9–11]. In bladder MRI, rFOV DWI showed better subjective image quality and superior diagnostic accuracy than full FOV DWI in differentiating NMIBC and MIBC [12–14]. However, compared with full FOV DWI, rFOV DWI had lower signal-to-noise ratio (SNR) because of smaller field of view. Moreover, it is worth noting that the use of rFOV DWI may result in longer scan time compared to conventional DWI [10, 13, 15], which poses a challenge to its routine clinical application.

Deep learning reconstruction (DLR) has emerged as a promising technique in medical imaging, particularly in body MRI. The application of DLR on T2-weighted imaging of the prostate, liver, and female pelvis has shown significant benefits, including shorter acquisition times and improved image quality compared to conventional reconstruction methods [16–19]. Recently, many studies have highlighted its potential for reduced scan time and improved image quality of DWI in liver, breast, and prostate evaluations [20–22]. We assumed that DLR could shorten the scanning time and improve image quality of rFOV DWI.

This study aimed to determine whether rFOV DWI with DLR can improve the accuracy of evaluating muscle invasion using VI-RADS.

## Materials and methods

### Participants

**Fig. 1:**
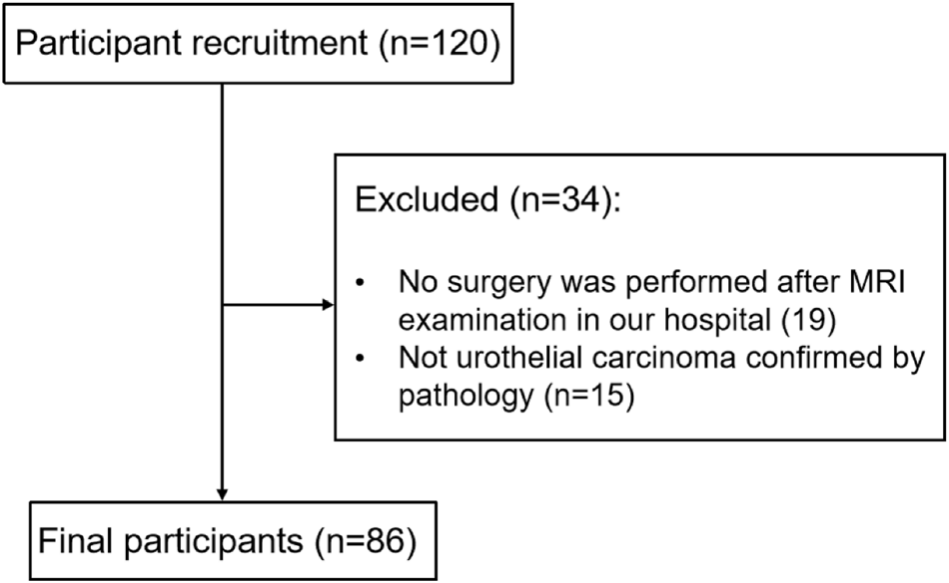
Flowchart shows the number of participants recruited and number and reason for exclusion from study

Participants with suspected bladder cancer who underwent a 3-T bladder MRI between August 2022 and February 2023 were consecutively enrolled. This observational prospective single-center study obtained Ethical approval. The study was conducted in line with the Declaration of Helsinki and its subsequent revisions, and written informed consent was obtained from all participants. Inclusion criteria were as follows: (1) Bladder tumor identified for the first time, with no prior treatment; (2) No bladder biopsy conducted within 2 weeks before MRI assessment; (3) Absence of contraindications for MRI examination. Exclusion criteria: (1) Patients who did not undergo surgery intervention; (2) Pathological confirmation of non-urothelial bladder cancer. The participant selection process is shown in Fig. 1. The data and material for this study are not available due to possible compromise of personal privacy.

### Image acquisition

All MRI examinations were performed on a 3-T MRI system (SIGNA Architect, GE Healthcare) with an AIR anterior array coil. Participants were instructed to void their bladders two hours before the imaging. For patients experiencing frequent urination, a water intake of 500–1000 mL was advised 30 min before the examination. Those without contraindications for spasmolytic treatment received a 1 mL intramuscular injection of scopolamine butylbromide.

The multiparameter MRI protocol included the following sequences: axial, coronal, and sagittal T2-weighted imaging (T2WI) sequence, axial fFOV DWI, standard rFOV (rFOV_STA_) DWI followed by fast rFOV with DLR (rFOV_DLR_) DWI with similar acquisition parameters and reduced numbers of excitation, axial dynamic contrast-enhanced imaging (DCEI). The *b*-values were 50 s/mm^2^ and 1000 s/mm^2^ for three DWIs. Apparent diffusion coefficient (ADC) maps were calculated for each DWI. The scan time of fFOV DWI, rFOV_STA_ DWI, and rFOV_DLR_ DWI were 1:39, 5:02, and 3:25 min, respectively. FOCUS DWI was performed as rFOV DWI in our study. Detailed image parameters and time are displayed in Table 1.

**Table 1:** MRI parameters for sequences

Parameter	Axial T2WI	Coronal T2WI	fFOV DWI	rFOV_DLR_ DWI	rFOV_STA_ DWI	DCEI
Repetition time (msec)	3846	4500	4500	5400	5400	3.5
Echo time (msec)	100	100	68.4–68.6	65.4–66.5	65.4–66.5	1.2
Field of view (mm)	230	250	360	240	240	360
Flip Angle	111	111	/		/	15
Matrix Size	416 × 320	352 × 320	128 × 96	140 × 70	140 × 70	226 × 224
Slice Thickness (mm)	4	4	4	4	4	1.6
Gap of slices	0	0	0.4	0.4	0.4	0
Number of Excitation and B value (s/mm^2^)	2	2	1 (50), 6 (1000)	1 (50), 5 (1000)	1 (50), 8 (1000)	1
Acquisition time (min: sec)	2:07	2:11	1:39	3:25	5:02	2:30

*DCEI* dynamic contrast-enhanced imaging, *DWI* diffusion-weighted imaging, *fFOV* full field-of-view, *rFOV*_*DLR*_ reduced field-of-view with deep learning reconstruction, *rFOV*_*STA*_ standard reduced field-of-view, *T2WI* T2 weighted imaging

The AIR^TM^ Recon DL algorithm (GE Healthcare) based on feedforward deep convolutional neural networks was used to reconstruct rFOV_DLR_ DWI. Convolutional neural networks accept raw unfiltered complex-valued input data and provide output images with improved signal-to-noise ratio [23]. The software provides a user-specified denoising level from 0% to 100%, where 0% means conventional reconstruction without DL; other options are as follows: low (33%), medium (50%), and high (75%). In the present study, a 75% noise reduction factor was chosen. The detailed network design and performance in phantom images are shown in the white paper [23].

### Image analysis

Two genitourinary radiologists (reader 1 Y.C., and reader 2 X.X.Z., with 29, and 4 years of experience in abdominal MRI, respectively) independently reviewed fFOV DWI, rFOV_STA_ DWI, and rFOV _DLR_ DWI in random order during separate sessions, with a month interval between sessions. All image analyses were performed on AW 4.7 workstation (GE Medical Systems). The presenter of the images (Y.C.W., with 9 years of experience in abdominal MR) recorded the reader’s rating results of imaging quality assessment and VI-RADS scoring. In cases with multiple lesions, the lesion with the greatest invasion depth or largest size (in cases of equal degrees of invasion) was selected by a radiologist (X.X.J., with 18 years of experience in abdominal MR) before assessment.

### Imaging quality assessment

Qualitative evaluation was performed using a 4-point scoring system. The evaluation criteria are as follows: overall image quality (1 = poor image quality; 2 = fair image quality; 3 = good image quality; 4 = excellent image quality), motion artifacts (1 = severe artifact with no diagnostic value; 2 = moderate artifact with effect on diagnostic assessment; 3 = mild artifact without interference of diagnostic assessment; 4 = no artifact), bladder wall sharpness (1 = severe blurring, 2 = intermediate blurring, 3 = slight blurring, 4 = no blurring).

For quantitative evaluation, oval regions of interest were manually drawn on the iliopsoas muscle and the lesion in a single representative slice of DWIs (*b* = 1000 s/mm^2^), and automatically copied to the ADC maps. The SNR of the lesion and the contrast-to-noise ratio (CNR) of the lesion to the iliopsoas muscle were calculated according to the following equations:

$${SNR}=\frac{\,{{SI}}_{{tumor}}}{{{SD}}_{{muscle}}}$$

$${CNR}=\frac{({{SI}}_{{tumor}}-{{SI}}_{{muscle}})}{\sqrt{({{{SD}}_{{tumor}}}^{2}+{{{SD}}_{{muscle}}}^{2})}}$$

SI_tumor_ and SD_tumor_ represent the mean and standard deviation values of signal intensity of the tumors respectively, while SI_muscle_ and SD_muscle_ represent the mean value and standard deviation of signal intensity of the iliopsoas muscle, respectively.

### Evaluation of muscular invasion by using VI-RADS

All MRI images were independently evaluated according to VI-RADS [3] by the above two readers without knowledge of the surgical or histologic findings. Category by single sequence (T2WI, fFOV DWI, rFOV_STA_ DWI, rFOV _DLR_ DWI, and DCEI) was separately assessed with an interval of two weeks between each sequence. And the final VI-RADS score of set1, set2, and set3 was assigned. Each set included axial, coronal, and sagittal T2W images, DCEI, and DWI with the corresponding ADC map. In detail, fFOV DWI was included in set 1, rFOV_STA_ DWI in set 2, and rFOV _DLR_ DWI in set 3. The 5-point scores using VI-RADS were compared with the pathological results of surgery.

### ADC values of bladder cancers

The ADC values were measured by using a single representative slice of the tumor. Regions of interest were manually drawn on fFOV DWI, and were copied to rFOV_STA_ DWI, and rFOV_DLR_ DWI with a *b* value of 1000 s/mm^2^, and the mean ADC of the ROI was recorded. Tumor stalk or thickened submucosa and vessels were excluded using T2WI as a reference.

### Reference standard

All patients underwent transurethral resection of bladder tumor or radical cystectomy within four weeks after MRI. When patients had both, radical cystectomy was considered as the final standard of reference. According to European association of Urology guidelines, a second TURB may be performed for high-risk patients [24].

The histological type, grade, and stage of the tumors were assessed by pathologists according to the 2016 World Health Organization grading systems and the 2017 American Joint Committee on Cancer/Union for International Cancer Control TNM staging system.

### Statistical analysis

The sample size of this study was calculated by comparing SNR and CNR means between fast sequence with DLR and standard sequence. A confidence interval of 95% and a power of 90% was considered. Details information on the sample size calculation and the tool used can be found in supplement S1 and Table S1 (supplement online). The number of patients needed in this study to obtain the desired power was 68.

The Kolmogorov–Smirnov test was used to test the normal distribution of quantitative data and Likert scales. This test showed that the distribution of the values of SNR, CNR, ADC value, and Likert scales of image quality were non-normal. Therefore, quantitative data and Likert scales were compared by Friedman test with Dunn’s pairwise post hoc test. Bonferroni correction *p* values for multiple comparisons were applied. Receiver operating characteristic curve analysis was used to analyze the accuracies of VI-RADS in predicting muscle invasion. The optimal cutoff value of the VI-RADS score was determined by maximization of Youden’s index. Sensitivity, specificity, positive predictive value, negative predictive value, accuracy, and area under the curve (AUC) were calculated for all radiologists. Delong’s test was used to calculate the difference between every two groups of AUC. Intraclass correlation coefficients were used to evaluate interobserver agreement for SNR, CNR, and ADC value. Additionally, interobserver agreements for qualitatively assessed image quality and VI-RADS score were evaluated through Cohen κ. The κ values were interpreted as follows: 0–0.20 = poor agreement, 0.21–0.40 = fair agreement, 0.41–0.60 = moderate agreement, 0.61–0.80 = substantial agreement, and 0.81–1 = excellent agreement. All statistical analyses were performed using the software SPSS version 27.0 (IBM). All tests were two-sided and statistical significance was determined to be *p* < 0.05.

## Results

### Participant characteristics

**Fig. 2:**
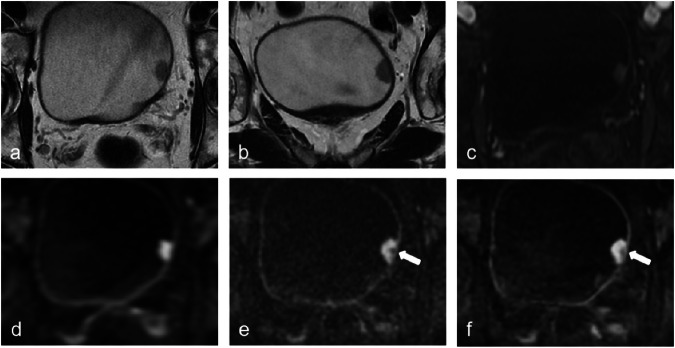
A 69-year-old man with pTa low-grade urothelial carcinoma. Axial (**a**) and coronal (**b**) T2WI show an exophytic tumor on the left side of bladder wall with no clear interruption of low-signal intensity muscularis propria, which was rated as T2 weighted category 3. DCEI (**c**) shows early enhancement of tumor, which was rated as DCEI category 3. fFOV DWI (**d**) shows a high signal-intensity tumor, which was rated as category 3. rFOV_STA_ DWI (**e**) and rFOV_DLR_ DWI (**f**) show high signal-intensity tumor with a low-signal-intensity stalk (arrow), which were rated as category 2. The VI-RADS score for set 1 was 3 for all readers, and the VI-RADS score for set 2 and set 3 was 2. DCEI, dynamic contrast-enhanced imaging; DWI, diffusion-weighted imaging; fFOV, full field-of-view; rFOV, reduced field-of-view; rFOV_DLR_, rFOV with DLR; T2WI, T2-weighted imaging

**Fig. 3:**
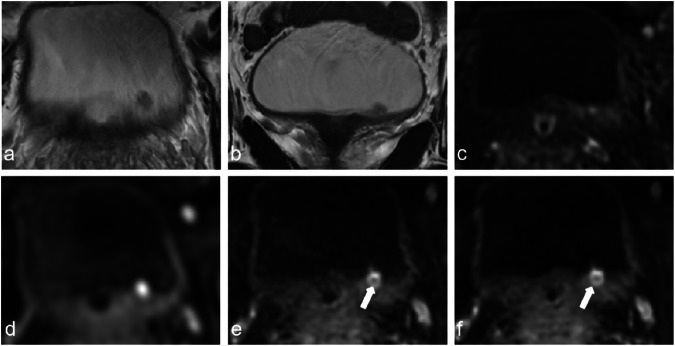
A 69-year-old man with pTa high-grade urothelial carcinoma. Axial (**a**) and coronal (**b**) T2WI show a small exophytic tumor on left lateral bladder wall with continuous low-signal intensity muscularis propria. DCE (**c**) image shows early enhancement of tumor. fFOV DWI (**d**) shows a high signal-intensity tumor without stalk. rFOV_STA_ DWI (**e**) and rFOV_DLR_ DWI (**f**) show high signal-intensity tumor with a low-signal-intensity stalk (arrow). All sequences were assigned category 1 and the VI-RADS score of all sets was 1. DCEI, dynamic contrast-enhanced imaging; DWI, diffusion-weighted imaging; fFOV, full field-of-view; rFOV, reduced field-of-view; rFOV_DLR_, rFOV with DLR; T2WI, T2-weighted imaging

Eighty-six participants (mean age ± standard deviation, 63 years ± 10; range, 39–82 years, 13 women) were successfully enrolled in the final analysis of our study. A total of 73 participants (85%) were diagnosed with NMIBC and the remaining 13 (15%) participants with MIBC. All participants underwent surgery within one month after MRI (range, 2–27 days). Table 2 summarizes the characteristics of both patients and focal lesions. Figures 2 and 3 show the representative lesions.

**Table 2:** Clinical and pathologic characteristics of patients (*n* = 86)

Characteristics	Value
Age, year	
Mean ± Standard Deviation	63 ± 10
Range	39–82
Gender	
Male	73 (85)
Female	13 (15)
Number of lesions	
Solitary	52 (60.0)
Multiple	34 (40.0)
Pathological T staging	
≤ T1	66 (77)
≥ T2	20 (23)
Histologic grade	
Low grade	34 (40)
High grade	52 (60)
Treatment method	
Radical cystectomy	9 (10)
TURBT	77 (90)

Numbers in parentheses are percentages

*TURBT* transurethral resection of bladder tumor

### Interobserver agreement

Cohen κ values for the categories of fFOV DWI, rFOV_STA_ DWI, rFOV _DLR_ DWI, and VI-RADS of different sets were in excellent agreement ranging from 0.92 to 0.95. Cohen κ values for qualitative image quality assessment demonstrated substantial to excellent agreement, ranging from 0.71 to 0.93. The intraclass correlation coefficients of SNR, CNR, and ADC value were substantial to excellent with values between 0.63 and 0.98, as summarized in Table S2.

### Imaging quality assessment

Detailed results for the qualitative image quality scores of the two readers for fFOV DWI, rFOV_STA_ DWI, and rFOV_DLR_ DWI are presented in Table 3. The overall image quality and sharpness were rated highest for rFOV_DLR_ DWI, followed by rFOV_STA_ DWI, and lowest for fFOV DWI (all *p* < 0.05). Concerning artifacts, significantly lower rating scores were assigned to fFOV DWI compared to rFOV_STA_ DWI and rFOV_DLR_ DWI (all *p* < 0.001), and no significant difference was found between rFOV_STA_ DWI and rFOV_DLR_ DWI (all *p* > 0.05).

**Table 3:** Qualitative image quality assessment of three DWIs

Reader	Parameters	fFOV DWI	rFOV_STA_ DWI	rFOV_DLR_ DWI	*p* value*
Reader 1	Overall image quality	2 (2, 2)	3 (3, 3)	4 (4, 4)	< 0.001
	Artifacts	3 (2, 3)	3 (3, 4)	3 (3, 4)	< 0.001
	Sharpness	2 (2, 3)	3 (3, 4)	4 (3, 4)	< 0.001
Reader 2	Overall image quality	2 (2, 2)	3 (3, 3)	4 (4, 4)	< 0.001
	Artifacts	3 (2, 3)	3 (3, 4)	3 (3, 4)	< 0.001
	Sharpness	2 (2, 3)	3 (3, 4)	4 (3.75, 4)	< 0.001

Data are medians with interquartile ranges in parentheses

*DWI* diffusion-weighted imaging, *fFOV* full field-of-view, *rFOV*_*DLR*_ reduced field-of-view with deep learning reconstruction, *rFOV*_*STA*_ standard reduced field-of-view

*p values were calculated across three imaging protocols

Table S3 shows the results of the quantitative evaluation of SNR and CNR. The SNR of the tumor was significantly lower with rFOV_STA_ DWI than with fFOV DWI (all *p* < 0.001). However, it was significantly increased by applying DLR to rFOV DWI (all *p* < 0.001). There was no significant difference in the SNR values between rFOV_DLR_ and fFOV DWI (all *p* > 0.05). The CNR between the tumor and iliopsoas muscle was higher for rFOV_DLR_ DWI than rFOV_STA_ DWI and fFOV DWI. There was no significant difference in the CNR values between rFOV_STA_ DWI and fFOV DWI (all *p* > 0.05).

The detailed *p*-value of pairwise comparison among the three sequences was shown in Table S4 in the Supplementary Material.

### The association between the ADC value and muscle infiltration

The fFOV DWI, rFOV_STA_ DWI, and rFOV_DLR_ DWI ADCs of all cases, NMIBC and MIBC are shown in Table 4. The differences in ADC values were significant between NMIBC and MIBC on fFOV DWI, rFOV_STA_ DWI and rFOV_DLR_ DWI (all *p* < 0.001). The mean ADC of all cases of rFOV_STA_ DWI and rFOV_DLR_ DWI was significantly lower than that of fFOV DWI (all *p* < 0.05). There was no significant difference between rFOV_STA_ DWI and rFOV_DLR_ DWI for all cases (all *p* > 0.05). The detailed *p*-value of pairwise comparison among the three sequences for ADC value was shown in Table S4 in the Supplementary Material.

**Table 4:** Comparison of ADC values between three DWIs

	ADC Values (× 10^−3^ mm^2^/sec)				*p* value		
Parameter	All case	NMIBC	MIBC	NMIBC vs MIBC	fFOV DWI vs rFOV_STA_ DWI of All cases	fFOV DWI vs rFOV_DLR_ DWI of All cases	rFOV_STA_ DWI vs rFOV_DLR_ DWI of All cases
Reader1							
fFOV DWI	1.26 (± 0.30)	1.35 (± 0.26)	0.96 (± 0.08)	< 0.01	0.01	< 0.001	0.76
rFOV_STA_ DWI	1.18 (± 0.26)	1.24 (± 0.24)	0.95 (± 0.14)	< 0.01			
rFOV_DLR_ DWI	1.18 (± 0.26)	1.27 (± 0.23)	0.91 (± 0.14)	< 0.01			
Reader2							
fFOVDWI	1.26 (± 0.30)	1.36 (± 0.26)	0.94 (± 0.08)	< 0.01	0.02	< 0.001	0.38
rFOV_STA_ DWI	1.18 (± 0.26)	1.25 (± 0.25)	0.94 (± 0.15)	< 0.01			
rFOV_DLR_ DWI	1.18 (± 0.26)	1.27 (± 0.23)	0.91 (± 0.13)	< 0.01			

*ADC* apparent diffusion coefficient, *DWI* diffusion-weighted imaging, *fFOV* full field-of-view, *rFOV*_*DLR*_ reduced field-of-view with deep learning reconstruction, *rFOV*_*STA*_ standard reduced field-of-view

^a^ Data are means ±  standard deviations

### Evaluation of muscular invasion by using VI-RADS

**Fig. 4:**
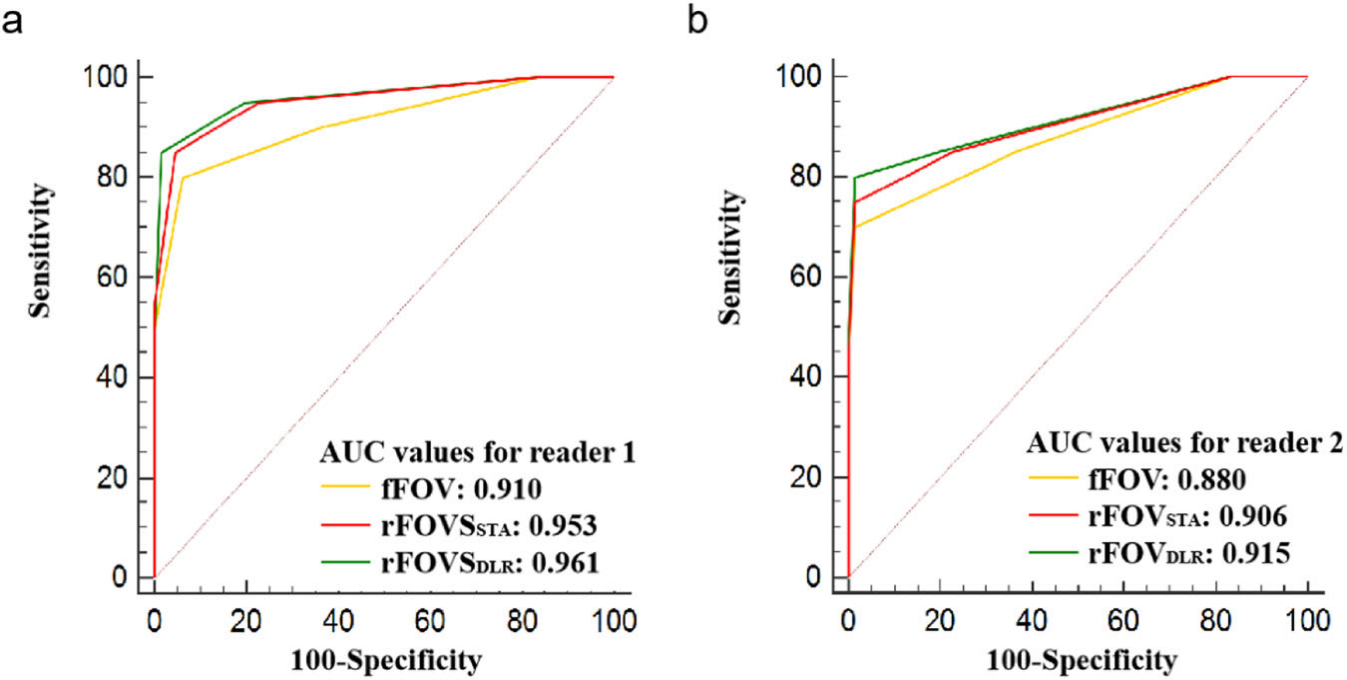
Comparison of ROC curves between categories by fFOV DWI, rFOV_STA_ DWI, and rFOV_DLR_ DWI for the evaluation of MIBC for reader 1 (**a**) and reader 2 (**b**). The optimal cutoff value for the category was 4. ROC, receiver operating characteristic; DWI, diffusion-weighted imaging; fFOV, single-shot; rFOV_DLR_, field-of-view optimized and constrained undistorted single-shot with deep learning reconstruction; rFOV_STA_, standard field-of-view optimized and constrained undistorted single-shot; MIBC, muscle-invasive bladder cancer

According to receiver operating characteristic (ROC) curve analysis, a score of 4 or greater was the cutoff value of categories of fFOV DWI, rFOV_STA_ DWI, rFOV_DLR_ DWI, and the final VI-RADS of set 1, set 2 and set 3 for both two readers. The diagnostic performance of categories by three DWIs and the final VI-RADS of set 1, set 2, and set 3 for evaluating muscle invasion was demonstrated in Table 5. The detailed *p*-value of pairwise comparison among the three sequences for AUC was shown in Table S5 in the Supplementary Material. Figure 4 shows receiver operating characteristic curve analyses of categories of fFOV DWI, rFOV DWI and rFOV_DLR_ DWI for diagnosing muscle invasion.

**Table 5:** Diagnostic performance of categories of three DWIs and VI-RADS score of three sets regarding the MIBC detection (cutoff value ≥ 4)

Parameter	fFOV DWI		rFOV_STA_ DWI		rFOV_DLR_ DWI		VI-RADS of set 1		VI-RADS of set 2		VI-RADS of set 3	
	Reader 1	Reader2	Reader 1	Reader 2	Reader 1	Reader 2	Reader 1	Reader 2	Reader 1	Reader 2	Reader 1	Reader 2
Sensitivity	80.0%	70.0%	85.0%	75.0%	85.0%	80.0%	85.0%	75.0%	85.0%	75.0%	85.0%	75.0%
Specificity	93.9%	98.5%	95.5%	98.5%	98.5%	98.5%	97.0%	98.5%	97.0%	98.5%	98.5%	98.5%
PPV	80.0%	93.3%	85.0%	93.8%	94.4%	94.1%	89.5%	93.8%	89.5%	93.8%	94.4%	93.8%
NPV	93.9%	92.9%	95.5%	92.9%	95.6%	94.2%	95.5%	92.9%	95.5%	92.9%	95.6%	92.9%
Accuracy	90.7%	91.9%	93.0%	93.0%	95.3%	94.2%	94.2%	93.0%	94.2%	93.0%	95.3%	93.0%
AUC	0.910	0.880	0.953	0.906	0.961	0.915	0.945	0.889	0.976	0.906	0.981	0.910
κ statistics	0.93		0.92	–	0.94	–	0.93	–	0.94	–	0.95	–

*AUC* area under the curve, *DWI* diffusion-weighted imaging, *fFOV* full field-of-view, *rFOV*_*DLR*_ reduced field-of-view with deep learning reconstruction, *rFOV*_*STA*_ standard reduced field-of-view, *VI-RADS* vesical imaging reporting and data system, *NPV* negative predictive value, *PPV* positive predictive value

The AUC for both rFOV_STA_ DWI and rFOV_DLR_ DWI were significantly higher than that of fFOV DWI (all *p* < 0.05). There was no significant difference in the AUC between rFOV_STA_ DWI and rFOV_DLR_ DWI (all *p* > 0.05). Notably, nine cases were rated 3 on the fFOV DWI but were 2 on rFOV_STA_ and rFOV_DLR_ DWI. Because, the stalks of nine tumors can be clearly displayed on reduced field-of-view (rFOV) DWI but not on full field-of-view (fFOV) DWI.

The AUC for VI-RADS of set 2 and set 3 were significantly higher than that of set 1 (all *p* < 0.05). There was no significant difference in the AUC between VI-RADS of set 2 and VI-RADS of set 3 (all *p* > 0.05).

## Discussion

Our results demonstrated that the diagnostic accuracy of rFOV DWI with DLR was better than fFOV DWI in evaluating muscle invasion of bladder cancer. And applying DLR on rFOV DWI could reduce scan time, and improve image quality while maintaining ADC value and diagnostic performance.

Compared to SS DWI, our findings have established the superior subjective image quality of rFOV_STA_ DWI, which was consistent with previous studies [13, 25]. In addition, our study further compared the SNR and CNR. Results showed that there was no significant difference in CNR between the two sequences. The SNR of rFOV_STA_ DWI was significantly lower than that of fFOV DWI. However, the application of DLR on rFOV DWI significantly improved image quality. Moreover, the scan time was reduced by 32% using this novel vendor-supplied DLR technology. This may be beneficial to routine clinical application of rFOV DWI. Consistent with our findings, many recent studies conducted using DLR technology on MRI of the abdomen, prostate, and female pelvis have achieved a reduction in scan time and an improvement in image quality [16–18].

In terms of diagnostic accuracy, we assessed and compared the diagnostic performance of three DWIs and the final VI-RADS scoring for three sets. Our results revealed that rFOV_STA_ and rFOV_DLR_ enabled better visualization of tumor stalks in night cases, which may be the reason for leading to the AUC for rFOV_STA_ and rFOV_DLR_ DWI being higher than SS DWI. Therefore, there was an improvement in AUC for rFOV_STA_ and rFOV_DLR_ DWI and VI-RADS of set 2 and set 3. In addition, there was no significant difference in AUC between rFOV_STA_ and rFOV_DLR_ DWI, suggesting the potential for DLR to maintain good diagnostic accuracy. These results strengthen the usefulness of incorporating rFOV_DLR_ DWI into clinical practice to further improve diagnostic accuracy. Because dynamic contrast-enhanced imaging is an important part of VI-RADS, we did not further analyze the effect of the combination rFOV DWI with T2WI on the diagnostic of “bi-parametric” MRI.

ADC value serves as a vital imaging biomarker for lesion detection, disease diagnosis, and prognostic assessment of treatment response in clinical practice. Our study evaluated the association between ADC values and muscle infiltration. Consistent with previous studies [12, 26], there were significant differences in ADC values between NMIBC and MIBC across all three DWI techniques, indicating that ADC values may serve as a reliable biomarker for distinguishing between NMIBC and MIBC in bladder cancer. For all cases, the ADC values of fFOV DWI were higher than those of rFOV DWI with and without DLR, which was also similar to a previous study [12]. This finding can be attributed to the improved clarity in delineating lesion boundaries on rFOV DWI, which reduced partial volume effects between the tumor and surrounding normal tissue [27]. This potentially improved the accuracy of ADC value measurements.

There are some limitations in our study. First, the sample size was relatively small, which might impact the statistical power of our results. Second, our study was a single-center research, all examinations were performed using a single 3-T MRI scanner with a commercially available DLR method. Therefore, larger multicenter studies are warranted. Third, we did not compare the effects of DLR on radiologists with different experience levels for evaluation of muscle invasion to further validate the clinical diagnostic applicability of DLR. We will further explore this content in future research.

In conclusion, rFOV DWI with DLR can improve diagnostic accuracy for evaluating muscle invasion. DLR would benefit the wide clinical application of rFOV DWI by reducing the acquisition time and improving the overall image quality while maintaining ADC value and diagnostic performance.

### Supplementary information

Supplementary Information

## Data Availability

The statement of ‘Availability of data and materials’ has been recorded in the first paragraph of the Materials and Methods section.

## Abbreviation

ADC: Apparent diffusion coefficient
AUC: Area under the curve
CNR: Contrast-to-noise ratio
DCEI: Dynamic contrast-enhanced imaging
DLR: Deep learning reconstruction
DWI: Diffusion-weighted imaging
fFOV: Full field-of-view
MIBC: Muscle-invasive bladder cancer
MRI: Magnetic resonance imaging
NMIBC: Non-muscle-invasive bladder cancer
rFOV: Reduced field-of-view
rFOV_DLR_: rFOV with DLR
ROC: Receiver operating characteristic
SNR: Signal-to-noise ratio
SS: Single-shot
T2WI: T2-weighted imaging
VI-RADS: Versical imaging reporting and data system
