# Supplementary material for: Reduced field-of-view DWI based on deep learning reconstruction improving diagnostic accuracy of VI-RADS for evaluating muscle invasion
Source: Insights Imaging. 2024 Jun 9;15:139. doi: 10.1186/s13244-024-01686-9 (PMC11162985; doi:10.1186/s13244-024-01686-9)
Supplement: Supplementary file 1 — Supplementary Information [file 13244_2024_1686_MOESM1_ESM.pdf]

Reduced Field-of-View DWI based on Deep Learning Reconstruction Improving Diagnostic Accuracy of VI-RADS for Evaluating Muscle Invasion

ELECTRONIC SUPPLEMENTARY MATERIAL

Supplement S1

According to the results of the previous research on the application of DLR on breast and liver DWI[1,2], we assume that the SNR and CNR between fast sequence with DL reconstruction and standard sequence will be significantly different. Therefore, sample size estimation was performed considering the difference of SNR and CNR between $rFOV_{DLR}$ DWI and $rFOV_{STA}$ DWI. The sample size calculation of this study was carried out after 30 patients were included due to no relevant research as reference. Reader 2 preliminarily measured and calculated the SNR and CNR of each sequence. The sample size is calculated by using the online tool (http://www.openepi.com/SampleSize/fFOVCC.htm) to select the model for comparing two means. The formula for sample size calculation for a two-sample t-test:

n = [2 * (Z_alpha/2 + Z_beta) ^2 * σ^2] / δ^2

Where: n is the required sample size per group. Z_alpha/2 is the critical value for a two-tailed test at the desired significance level (0.05 was considered in the study), which corresponds to the proportion of data falling in the tails of the distribution. Z_beta is the critical value corresponding to the desired statistical power (0.9 was considered in our study), representing the probability of correctly rejecting the null hypothesis when the alternative hypothesis is true. σ is the standard deviation of the outcome variable. δ is the minimum meaningful difference in means that you want to detect. The calculation results are shown in Table S1. The number of patients needed in this study to obtain the desired power was 68.

Supplementary Table 1. Sample Size Calculation by Quantitative Image Quality Assessment of rFOV DWI			
	$rFOV_{STA}$ DWI	$rFOV_{DLR}$ DWI	Sample Size
SNR	52.6±15.6	77.6±29.7	19
CNR	6.3±1.4	7.2±1.8	68

Note. Data are means ± standard deviation. CNR = contrast noise ratio; DWI = diffusion-weighted imaging; fFOV = full field-of-view; $rFOV_{DLR}$ = reduced field-of-view with deep learning reconstruction; $rFOV_{STA}$ = standard reduced field-of-view; SNR = signal noise ratio.

Supplementary Table 2. Interobserver variability of qualitative imaging quality assessment, SNR, CNR and ADC value between fFOV DWI, $rFOV_{STA}$ DWI, and $rFOV_{DLR}$ DWI

Parameters	fFOV DWI	$rFOV_{STA}$ DWI	$rFOV_{DLR}$ DWI
Overall image quality	0.71	0.75	0.78
Artifacts	0.84	0.84	0.84
Sharpness	0.83	0.93	0.88
SNR	0.73	0.63	0.75
CNR	0.86	0.83	0.92
ADC value	0.96	0.97	0.98

ADC = apparent diffusion coefficients; CNR = contrast noise ratio; DWI = diffusion-weighted imaging; fFOV = full field-of-view; $rFOV_{DLR}$ = reduced field-of-view with deep learning reconstruction; $rFOV_{STA}$ = standard reduced field-of-view; SNR = signal-to-noise ratio.

Supplementary Table 3. Comparison of SNR and CNR for fFOV DWI, $rFOV_{STA}$ DWI and $rFOV_{DLR}$ DWI

Reader	Parameter	fFOV DWI	$rFOV_{STA}$ DWI	$rFOV_{DLR}$ DWI	*P* value*
Reader 1	SNR	102.7±53.1	55.6±21.7	87.2±39.2	＜0.001
	CNR	6.3±1.8	6.7±1.9	7.5±2.3	＜0.05
Reader 2	SNR	98.5±43.7	52.1±19.6	96.6±55.7	＜0.001
	CNR	6.4±2.1	6.6±2.0	7.5±2.4	＜0.05

Note—Data are means ± standard deviations. * P values were calculated across three imaging protocols.

CNR= contrast noise ratio; DWI = diffusion-weighted imaging; fFOV = full field-of-view; $rFOV_{DLR}$ = reduced field-of-view with deep learning reconstruction; $rFOV_{STA}$ = standard reduced field-of-view.

Supplementary Table 4. P values of post-hoc analysis of subjective image quality scores, SNR, CNR, and ADC value for pairwise comparison of fFOV DWI, $rFOV_{STA}$ DWI, and $rFOV_{DLR}$ DWI

	P value of fFOV DWI vs. $rFOV_{STA}$ DWI	P value of fFOV DWI vs. $rFOV_{DLR}$ DWI	P value of $rFOV_{STA}$ DWI vs. $rFOV_{DLR}$ DWI
Reader 1			
Overall image quality	<0.001	<0.001	<0.001
Artifacts	<0.001	<0.001	0.42
sharpness	0.007	<0.001	0.03
SNR	<0.001	0.28	<0.001
CNR	> .99	<0.001	<0.001
ADC value	0.01	<0.001	> .99
Reader 2			
Overall image quality	<0.001	<0.001	<0.001
Artifacts	<0.001	<0.001	0.34
sharpness	<0.001	<0.001	0.04
SNR	<0.001	> .99	<0.001
CNR	0.97	<0.001	<0.001
ADC value	0.03	<0.001	0.83

ADC = apparent diffusion coefficients; CNR = contrast noise ratio; DWI = diffusion-weighted imaging; fFOV = full field-of-view; $rFOV_{DLR}$ = reduced field-of-view with deep learning reconstruction; $rFOV_{STA}$ = standard reduced field-of-view.

Supplementary Table 5. Results of AUC for pairwise comparison of three DWIs and VI-RADS of three sets.

Parameters	P value
Reader 1 (NMIBC)	
fFOV DWI vs. $rFOV_{STA}$ DWI	0.04
fFOV DWI vs. $rFOV_{DLR}$ DWI	0.03
$rFOV_{STA}$ DWI vs. $rFOV_{DLR}$ DWI	0.14
Set1 vs. Set2	0.03
Set1 vs. Set3	0.02
Set2 vs. Set3	0.21
Reader 2 (NMIBC)	
fFOV DWI vs. $rFOV_{STA}$ DWI	0.03
fFOV DWI vs. $rFOV_{DLR}$ DWI	0.02
$rFOV_{STA}$ DWI vs. $rFOV_{DLR}$ DWI	0.15
Set1 vs. Set2	0.04
Set1 vs. Set3	0.04
Set2 vs. Set3	0.28

AUC = area under the curve; DWI = diffusion-weighted imaging; fFOV = full field-of-view; $rFOV_{DLR}$ = reduced field-of-view with deep learning reconstruction; $rFOV_{STA}$ = standard reduced field-of-view; VI-RADS = vertical imaging reporting and data system

1 Kim DH, Kim B, Lee H-S et al (2023) Deep Learning-Accelerated Liver Diffusion-Weighted Imaging: Intraindividual Comparison and Additional Phantom Study of Free-Breathing and Respiratory-Triggering Acquisitions. Invest Radiol. 10.1097/RLI.0000000000000988

2 Lee EJ, Chang Y-W, Sung JK, Thomas B (2022) Feasibility of deep learning k-space-to-image reconstruction for diffusion weighted imaging in patients with breast cancers: Focus on image quality and reduced scan time. Eur J Radiol 157:110608
